# Mathematical Models to Measure the Variability of Nodes and Networks in Team Sports

**DOI:** 10.3390/e23081072

**Published:** 2021-08-19

**Authors:** Fernando Martins, Ricardo Gomes, Vasco Lopes, Frutuoso Silva, Rui Mendes

**Affiliations:** 1 Instituto Politécnico de Coimbra, ESEC, UNICID-ASSERT, 3030-329 Coimbra, Portugal; fmlmartins@esec.pt (F.M.); rmendes@esec.pt (R.M.); 2 Instituto de Telecomunicações, Delegação da Covilhã, 6201-001 Covilhã, Portugal; fsilva@di.ubi.pt; 3 Instituto Politécnico de Coimbra, IIA, ROBOCORP, 3030-329 Coimbra, Portugal; 4 CIDAF, FCDEF, Universidade de Coimbra, 3040-248 Coimbra, Portugal; 5 Department of Informatics, Universidade da Beira Interior, 6201-001 Covilhã, Portugal; vasco.lopes@ubi.pt

**Keywords:** entropy, football, social network analysis, Markov chain, performance analysis, dynamical systems

## Abstract

Pattern analysis is a widely researched topic in team sports performance analysis, using information theory as a conceptual framework. Bayesian methods are also used in this research field, but the association between these two is being developed. The aim of this paper is to present new mathematical concepts that are based on information and probability theory and can be applied to network analysis in Team Sports. These results are based on the transition matrices of the Markov chain, associated with the adjacency matrices of a network with *n* nodes and allowing for a more robust analysis of the variability of interactions in team sports. The proposed models refer to individual and collective rates and indexes of total variability between players and teams as well as the overall passing capacity of a network, all of which are demonstrated in the UEFA 2020/2021 Champions League Final.

## 1. Introduction

Pattern analysis is a well-established research topic in team sports performance analysis, with dyadic interactions between players of the same team being represented in the form of passes [1,2,3,4]. A common goal in the many metrics used to describe the individual and group behavior in team sports is the identification of the most influential players or a depiction of the team’s behavior in terms of passing interactions, allowing for a characterization of the team’s organization along with the identification of the roles each player has in the network [1,2,5,6]. 

Metrics such as degree centrality, closeness, betweenness and the eigenvector are often used to analyse social interactions in a wide variety of activities ranging from the analysis of social media networks [7,8] to team sports match performance analysis in sports such as volleyball [9,10], handball [11], rugby [12] or football [2,5,6,13,14,15], among others. 

Broadly, these metrics attempt to identify the central elements in a group, the elements with more connections within it or the most influential ones [5,7]. In team sports such as football, knowing the most influential player or the player who is the element with more connections with the remaining elements of the team is crucial, as the opposing team may create strategies to prevent the ball from reaching said players, therefore creating difficulties for the opposing team [1,2,3,5,6]. 

The application of the concept of entropy to model the interactions of players passing the ball in football has also been used [16,17,18], pointing to higher entropy values leading to greater chances of creating goal-scoring opportunities [17]. The importance of entropy in football lies in the recognition that variability in the behavioral patterns of interaction between players is beneficial for the team, as the system is more unpredictable. Here, unpredictability leads to greater difficulties for the opposing team to interrupt the passing sequences.

An alternative approach to the analysis of these passing sequences in football has also been used, where the predictability of the passes being made is determined using Markov chains [19,20] and, associated to these, node and network entropy mathematical models [21]. These have attempted to explain the degree of variability associated with conditional probabilities, adding a non-linear perspective to a Bayesian approach to match performance analysis and providing a prediction of the variability of occurrence of passes. However, the individual and collective capacity of variability is not given, nor it is possible to assess the level of variability in a comparable ratio and index. Knowing how much variability of a node (player) or a system (team) is situational and specific to each condition (game), neither allows for a clear view of the level of performance that may be attainable, nor of the comparison between different conditions within or between games. 

Based in a mixed Bayesian and non-linear approach to team sports analysis, this work aims to introduce new individual and collective rates and indexes of variability within the team, providing new insights about the passing and receiving capacity of a certain team. These new mathematical models allow for a bounded analysis of the passing and receiving capacity of variability, allowing for the comparison between different players, between games or even between different tactical formations during the game, providing valuable information about the individual and team performance in various contexts of the game. 

## 2. Variability of Nodes and Networks

Based on information theory and probability theory, this section presents mathematical concepts that can be applied to networks that are considered weighted digraphs or weighted directed networks. The elementary concepts of nodes and networks used in this paper are presented in [5,22,23]. So, we assume that $A_{D}^{w}$. is the weighted adjacency matrix of a weighted digraph with *n* nodes, $G_{D}^{w}$. Therefore, we consider the concepts transition matrix of the Markov chain, $M_{T}$, associated with $A_{D}^{w}$ of a $G_{D}^{w}$, the k-step node transition and the probability of all nodes in the network after k times steps presented in [21]. 

Considering the mathematical concept of the entropy of a random variable, defined on the sample space of a random experiment and taking on a finite number [24] (p. 51), [25] and [26] (p. 19), and a weighted digraph with *n* nodes, we define the concept of *rate of passing of a node*, and it is the rate of possible variability in passing from this node to all other nodes of a network.

**Definition** **1.** *Given a weighted digraph,*$G_{D}^{w}$*, with n nodes. The*$R_{i}^{out}\left(n_{i}\right)$*is called rate of passing of a node*$n_{i}$*and it is the rate of possible variability of a node, in passing from node*$n_{i}$*to all other nodes*$n_{j}$. *It is determined by:*

(1) $$R_{i}^{out}\left(n_{i}\right)=-\overset{n}{\underset{j=1}{\sum }}\left(\frac{\sum_{j=1}^{n}w_{ij}}{L_{D}^{w}}\times m_{ij}{log}_{2}m_{ij}\right)\;$$

*where*$m_{ij}$*are the elements of*$M_{T}$*associated with*$A_{D}^{w}$*of*$G_{D}^{w}$, i,j=1,…,n.

**Remark** **1.** *In Definition 1, if we replace*$w_{ij}$*by*$w_{ji}$*and*$m_{ij}$*by*$m_{ji}$*, we obtain the concept rate of reception,*$R_{i}^{in}\left(n_{i}\right)$*, i.e., the capacity of the variability of a node,when a reception by*$n_{i}$*from all other nodes*$n_{j}$*can occur*.

Based on the mathematical concept of the rate transmission [25] and a weighted digraph with *n* nodes, we can propose the concept of the *index rate of passing of a node*.

**Proposition** **1.** *Given a weighted digraph,* $G_{D}^{w}$ *, with n nodes, the* $IndR_{i}^{out}\left(n_{i}\right)$ *, index of rate of passing of a node* $n_{i}$ *, is determined by:*

(2) $$IndR_{i}^{out}\left(n_{i}\right)=\frac{R_{i}^{out}\left(n_{i}\right)}{{log}_{2}n}.$$

**Proof.**  Consider that the index of rate of passing of a node $n_{i}$ is the ratio between $R_{i}^{out}\left(n_{i}\right)$ and the maximum that $R_{i}^{out}\left(n_{i}\right)$ can assume. Thus,

(3) $$IndR_{i}^{out}\left(n_{i}\right)=\frac{R_{i}^{out}\left(n_{i}\right)}{max\left(-\sum_{j=1}^{n}\left(\frac{\sum_{j=1}^{n}w_{ij}}{L_{D}^{w}}\times m_{ij}{log}_{2}m_{ij}\right)\right)}.$$

By Shannon [25] (p. 394), the maximum value occurs when $m_{ij}=\frac{1}{n}$. Then,$\;max\left(-\overset{n}{\underset{j=1}{\sum }}\left(\frac{\sum_{j=1}^{n}w_{ij}}{L_{D}^{w}}\times m_{ij}{log}_{2}m_{ij}\right)\right)={log}_{2}n$.

Therefore,

(4) $$\frac{R_{i}^{out}\left(n_{i}\right)}{max\left(-\sum_{j=1}^{n}\left(\frac{\sum_{j=1}^{n}w_{ij}}{L_{D}^{w}}\times m_{ij}{log}_{2}m_{ij}\right)\right)}=\frac{R_{i}^{out}\left(n_{i}\right)}{{log}_{2}n}.$$

□

**Remark** **2.** *In Proposition 1, if we consider*$R_{i}^{in}\left(n_{i}\right)$*, we obtain the concept of the index of rate of reception of a node*$n_{i}$*,*$IndR_{i}^{in}\left(n_{i}\right)$.

Considering the mathematical concept of the *rate of passing of a node* and the rate of variability when a pass can occur from this node to all other nodes of a network, we define the concept of the *rate of passing of a network.*

**Definition** **2.** *Given a weighted digraph,*$G_{D}^{w}$*, with n nodes, the*$R_{N}^{out}$*is called rate of passing of a network, and it is the rate of variability of a network, when a pass from node*$n_{i}$*to all other nodes*$n_{j}$*can occur. It is determined by:*

(5) $$R_{N}^{out}=\sum_{i=1}^{n}R_{i}^{out}\left(n_{i}\right),$$

*where*$m_{ij}$*are the elements of*$M_{T}$*associated with the*$A_{D}^{w}$*of*$G_{D}^{w}$, i,j=1,…,n.

**Remark** **3.** *In Definition 2, if we replace*$w_{ij}$*with*$w_{ji}$*and*$m_{ij}$*with*$m_{ji}$*, we obtain the concept of the rate of reception,*$R_{N}^{in}$*, i.e., the capacity of the variability of a network, when a reception can occur by*$n_{i}$*from all other nodes*$n_{j}$.

Based on the mathematical concept of the rate reception [25] and a weighted digraph with *n* nodes, we can propose the concept of the *index rate of passing of a network*.

**Proposition** **2.** *Given a weighted digraph,* $G_{D}^{w}$ *, with n nodes, the* $IndR_{N}^{out}$ *, index of rate of passing of a network, is determined by:*

(6) $$IndR_{N}^{out}=\frac{R_{N}^{out}}{{log}_{2}n}.$$

**Proof.**  Consider that the index of rate of passing of a node $n_{i}$ is the ratio between $R_{i}^{out}\left(n_{i}\right)$ and the maximum that $R_{i}^{out}\left(n_{i}\right)$ can assume. Thus,

(7) $$IndR_{N}^{out}=\frac{R_{N}^{out}}{max\left(-\sum_{i=1}^{n}\sum_{j=1}^{n}\left(\frac{\sum_{j=1}^{n}w_{ij}}{L_{D}^{w}}\times m_{ij}{log}_{2}m_{ij}\right)\right)}.$$

By Shannon [25] (p. 394), the maximum value occurs when all probabilities are equiprobable. Then, $max\left(-\overset{n}{\underset{i=1}{\sum }}\overset{n}{\underset{j=1}{\sum }}\left(\frac{\sum_{j=1}^{n}w_{ij}}{L_{D}^{w}}\times m_{ij}{log}_{2}m_{ij}\right)\right)={log}_{2}n$.

Therefore,

(8) $$\frac{R_{N}^{out}}{max\left(-\sum_{i=1}^{n}\sum_{j=1}^{n}\left(\frac{\sum_{j=1}^{n}w_{ij}}{L_{D}^{w}}\times m_{ij}{log}_{2}m_{ij}\right)\right)}=\frac{R_{N}^{out}}{{log}_{2}n}.$$

□

**Remark** **4.** *In Proposition 2, if we consider*$R_{N}^{in}$*, we obtain the concept of the index of rate of reception of a network,*$IndR_{N}^{in}$.

Based on the mathematical concept of the entropy of a random variable *X* with marginal distribution of joint distribution *(X,Y)* of the Markov Chain associated with the weighted adjacency matrix of a weighted digraph with *n* nodes [25] (p. 395), we can define the concept of the *rate of total variability between nodes that performed the passing in a network*.

**Proposition** **3.** *Given a weighted digraph,*$G_{D}^{w}$*, with n nodes and two random variables X and Y such that the pair of transmitter and receiver (X,Y) is the joint distribution of the Markov Chain associated with*$A_{D}^{w}$*of*$G_{D}^{w}$*, the*$E_{N}^{out}$*is called the rate of total out-entropy of a network. It is the rate of total variability between nodes that performed the passing in a network, determined by:*

(9) $$E_{N}^{out}=-\sum_{i=1}^{n}\sum_{j=1}^{n}\left(\frac{w_{ij}}{L_{D}^{w}}\right){log}_{2}\left(\sum_{j=1}^{n}\frac{w_{ij}}{L_{D}^{w}}\right),$$

*where*$w_{ij}$*are the elements of*$A_{D}^{w}$*and*$L_{D}^{w}=\overset{n}{\underset{i=1}{\sum }}\overset{n}{\underset{j=1}{\sum }}w_{ji}$*,*i,j=1,…,n.

**Proof.**  By the notion of marginal distribution in *X* of a joint distribution [25] (p. 395), we obtain

(10) $$E_{N}^{out}=-\sum_{i=1}^{n}p\left(X=x_{i}\right){log}_{2}p\left(X=x_{i}\right)\;=-\sum_{i=1}^{n}\sum_{j=1}^{n}p\left(X=x_{i},\;Y=y_{j}\right)log_{2}\left(\sum_{j=1}^{n}p\left(X=x_{i},\;Y=y_{j}\right)\right)=-\sum_{i=1}^{n}\sum_{j=1}^{n}\left(\frac{w_{ij}}{L_{D}^{w}}\right){log}_{2}\left(\sum_{j=1}^{n}\frac{w_{ij}}{L_{D}^{w}}\right).$$

□

**Remark** **5.** *In Proposition 3, if we consider the mathematical concept of the entropy of a random variable Y with marginal distribution of joint distribution (X,Y) of the Markov Chain associated with the weighted adjacency matrix of a weighted digraph with n nodes [25] (p. 395), we can obtain the concept of the rate of total in-entropy of a network,*$E_{N}^{in}$.

Based on the mathematical concept of *rate of mutual information* between two random variables *X* and *Y* such that the pair of transmitter and receiver *(X,Y)* is the joint distribution of the Markov Chain associated with the weighted adjacency matrix of a weighted digraph with *n* nodes [26] (p. 31), [27] (p. 246), we can propose the concept of the *capacity of passing of network* when we know information about the receivers.

**Proposition** **4.** *Given a weighted digraph,*$G_{D}^{w}$*, with n nodes and two random variables X and Y such that the pair of transmitter and receiver (X,Y) is the joint distribution of the Markov Chain associated with*$A_{D}^{w}$*of*$G_{D}^{w}$*, the*$C_{N}^{out}$*is the capacity of passing of a network and is determined by:*

(11) $$C_{N}^{out}=E_{N}^{out}-R_{N}^{in}$$

*where*i,j=1,…,n.

**Proof.**  Consider two random variables *X* and *Y* such that the pair of transmitter and receiver *(X,Y)* is the joint distribution of the Markov Chain associated with $A_{D}^{w}$ of $G_{D}^{w}$, i,j=1,…,n.

By concept of rate transmission presented in Shannon [25] (p. 407), we obtain

(12) $$C_{N}^{out}=-\sum_{i=1}^{n}\sum_{j=1}^{n}p\left(X=x_{i},\;Y=y_{j}\right)log_{2}\left(\sum_{j=1}^{n}p\left(X=x_{i},\;Y=y_{j}\right)\right)\;+\sum_{i=1}^{n}\sum_{j=1}^{n}p\left(X=x_{i},\;Y=y_{j}\right)log_{2}\left(p\left(X=x_{i}|\;Y=y_{j}\right)\right)=\;-\sum_{i=1}^{n}\sum_{j=1}^{n}\left(\frac{w_{ij}}{L_{D}^{w}}\right){log}_{2}\left(\sum_{j=1}^{n}\frac{w_{ij}}{L_{D}^{w}}\right)+\sum_{j=1}^{n}p\left(Y=y_{j}\right)\sum_{i=1}^{n}m_{ij}{log}_{2}\left(m_{ij}\right)=E_{N}^{out}-\;R_{N}^{in}.$$

□

**Remark** **6.** *Similarly to Proposition 4, considering the concept of the capacity of reception* [25] *and a weighted digraph with n nodes, we can propose the concept of the capacity of reception of a network,* $C_{N}^{in}=E_{N}^{in}-R_{N}^{out}$.

Based on the mathematical concept of the rate of transmission [25] and a weighted digraph with *n* nodes, we can propose the concept of the index of capacity of passing of a network.

**Proposition** **5.** *Given a weighted digraph,*$G_{D}^{w}$*, with n nodes and two random variables X and Y such that the pair of transmitter and receiver (X,Y) is the joint distribution of the Markov Chain associated with*$A_{D}^{w}$*of*$G_{D}^{w}$*, the*$IndC_{N}^{out}$*is the index of capacity of passing of a network and is determined by:*

(13) $$IndC_{N}^{out}=\left|\frac{C_{N}^{out}}{{log}_{2}n}\right|,$$

*where*$m_{ij}$*are the elements of*$M_{T}$*associated with*$A_{D}^{w}$*,*i,j=1,…,n.

**Proof.**  Consider that the index of rate of passing of a network is the ratio between $C_{N}^{out}$ and the maximum that $C_{N}^{out}$ can assume and two random variables *X* and *Y* such that the pair of transmitter and receiver *(X,Y)* is the joint distribution of the Markov Chain associated with $A_{D}^{w}$ of $G_{D}^{w}$, i,j=1, …, n. Thus,

(14) $$IndC_{N}^{out}=\left|\frac{C_{N}^{out}}{\underset{}{\max}C_{N}^{out}}\right|.$$

By Shannon [25] (p. 417), we obtain

(15) $$\underset{}{\max}C_{N}^{out}=\underset{}{\max}\left[-\sum_{i=1}^{n}\sum_{j=1}^{n}p\left(X=x_{i},\;Y=y_{j}\right)log_{2}\left(\sum_{j=1}^{n}p\left(X=x_{i},\;Y=y_{j}\right)\right)+\sum_{i=1}^{n}\sum_{j=1}^{n}p\left(X=x_{i},\;Y=y_{j}\right)log_{2}\left(p\left(X=x_{i}|\;Y=y_{j}\right)\right)\right]\;\leq \underset{}{\max}\left[-\sum_{i=1}^{n}\sum_{j=1}^{n}p\left(X=x_{i},\;Y=y_{j}\right)log_{2}\left(\sum_{j=1}^{n}p\left(X=x_{i},\;Y=y_{j}\right)\right)\right]={log}_{2}n.$$

So,

(16) $$IndC_{N}^{out}=\left|\frac{C_{N}^{out}}{{log}_{2}n}\right|.$$

□

**Remark** **7.** *In Proposition 5, if we consider*$C_{N}^{in}$*, we obtain theindex of capacity of reception of a network,*$IndC_{N}^{in}$.

## 3. Experimental Results

The 2020/2021 Champions League final was used to demonstrate and interpret the mathematical models proposed. The two opposing teams were Manchester City (MC) and Chelsea (CH). The match, after being originally scheduled to be played in Instambul, Turkey, took place in Porto, Portugal due to the COVID-19 pandemic restrictions at the time. In a very levelled match, CH won 1–0, with Kay Havertz scoring the winning goal. For the notational analysis of each transition between nodes, i.e., passing sequence, the uPATO platform was used [22,28]. The adjacency matrices were computed to calculate all transition state matrices, as described in [21].

The CH starting team were: Édouard Mendy (1); Ben Chilwell (2); César Azpilicueta (3); Thiago Silva (4); Antonio Rüdiger (5); Jorginho (6); Reece James (7); N’Golo Kanté (8); Timo Werner (9); Mason Mount (10); and Kay Havertz (11). Substitutes Andreas Christensen (12), Mateo Kovacic (13) and Christian Pulisic (14) entered the game at minutes 39, 66 and 80, with Thiago Silva (4), Mason Mount (10) and Timo Werner (9) coming out, respectively. The MC starting eleven were: Ederson (1); Kyle Walker (2); John Stones (3); Rúben Dias (4); Oleksandr Zinchenko (5); Ilkay Gundogan (6); Bernardo Silva (7); Phill Foden (8); Kevin de Bruyne (9); Raheem Sterling (10); and Riyad Mahrez (11). Used substitutes were Gabriel Jesus (12), who replaced Kevin de Bruyne (9) at minute 60; Sergio Aguero (13), who entered the pitch for Sterling (10) at minute 77; and Fernandinho (14), who substituted Silva (7) at minute 60.

**Figure 1:**
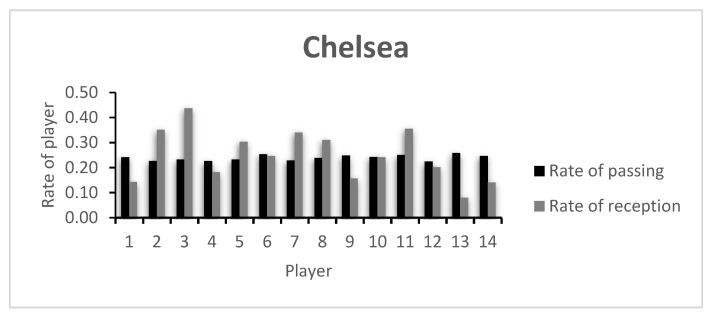
Chelsea’s rate of passing and reception.

**Figure 2:**
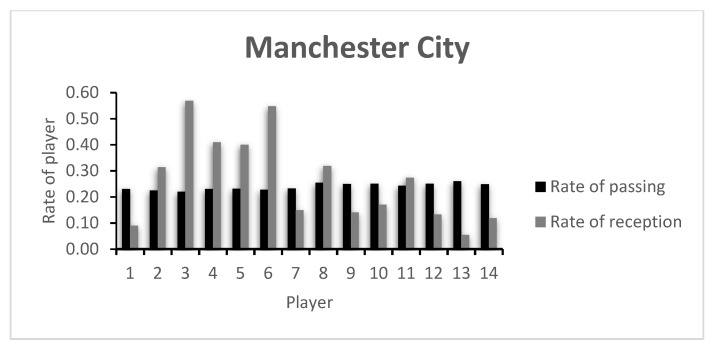
Mancester City’s Rate of passing and reception.

The rate of passing variability and receiving variability of each player on both teams (CH and MC, respectively) is presented in Figure 1 and Figure 2, and in Table A1 of the Appendix A. The players of both teams are relatively uniform and show little capacity of variability, showing that the teams have well established passing patterns in the game and that no player stands out in this regard.

When looking at the capacity of receiving variability, Player 1 is among the teams’ least variable. These are both goalkeepers, and the obtained values are in accordance with what is expected in a football game: passing to the goalkeeper is not common and very often regarded as an extraordinary occasion. Alternatively, and if this player takes part in the organization of the attack, they receive the ball from a very limited number of players—usually the center backs. In fact, this kind of option is taken in MC’s strategy when the team incorporates the goalkeeper in the passing sequences. Naturally, variability here is minimal. It is also worth noting that field players with little capacity of receiving variability such as CH’s Player 4 (Thiago Silva) or the substitute Players 13 and 14 (Kovacic and Pulisic) may show this characteristic due to the time each player played. Thiago Silva played only 39 min due to a sudden injury, and Kovacic and Pulisic entered late in the game. As for Player 9 (Timo Werner), the fact that this is a forward/striker, the early lead by CH in the game, or the team’s tactical setup may explain the low value.

Regarding MC’s metrics, and apart from Player 1, Players 7, 9 and 10 and the substitutes 12, 13 and 14 had low receiving variability capacity. Interestingly, these were the players that were substituted during the match. This may show that the technical staff were right to be dissatisfied with the players’ performance and attempted to change the course of the game.

In practical terms, knowing that a player has a higher rate of variability may indicate that he shows a higher degree of unpredictability, and it therefore is more difficult to predict to where the ball is going to go. The opposing team will face greater challenges when playing against unpredictable players. On the other hand, variability must also be seen within the group interactions. For example, teammates may have difficulties in creating synergies with highly variable players due to their intrinsic higher unpredictability. There is, therefore, a thin balance between what can be predictable and bring stability and reliability to the game, and the variable, unpredictable and creative.

It is important to note that the units of the rate of variability metric are arbitrary and are related exclusively to each node’s maximum capacity, making comparisons between nodes (players) difficult. For a more comparable analysis, the index rate of variability levels down all values and places them between 0 (least variability) and 1 (highest variability).

**Figure 3:**
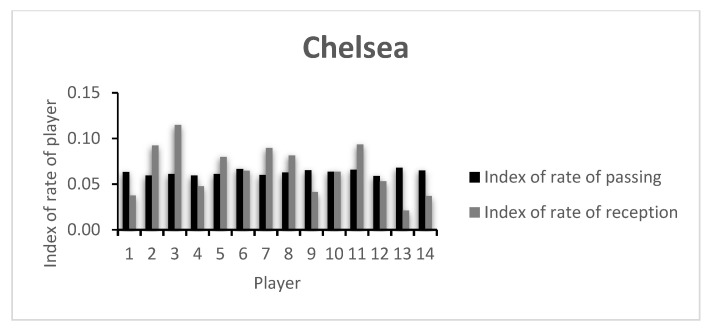
Chelsea’s index of rate of passing and reception.

**Figure 4:**
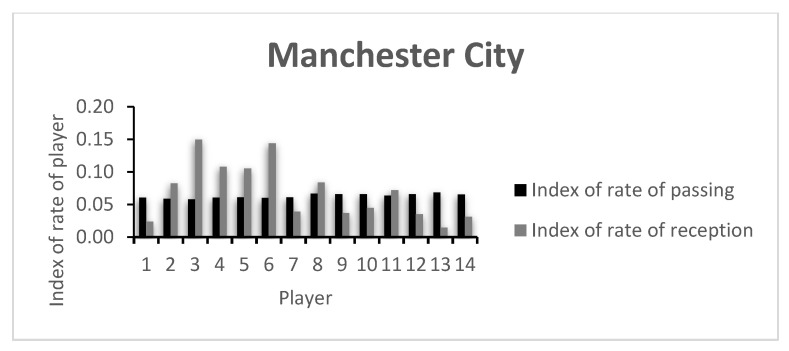
Mancester City’s index of rate of passing and reception.

The index of rate of passing and receiving for each player that participated in the game are presented in Figure 3 and Figure 4. The values of each player are presented in Table A2 of the Appendix A.

The low index values presented by the players are in accordance with the rate of variability presented in Figure 1 and Figure 2, as well as in Table A1. Here, a comparative analysis may be made between players, taking into consideration that the values shown are always between 0 and 1, the least and most variability possible, respectively. In this example, both teams keep relatively similar values for the index of passing variability. However, the index of receiving variability shows more dissimilar values, with CH’s Player 3 presenting the highest team value. CH’s Player 4 was substituted early in the game, showing a low index value. An analysis of the opposing team shows that apart from MC’s goalkeeper (Player 1), Players 7 and 9, both on the starting 11 and both substituted around minute 60, showed low indexes of reception variability. This may have happened due to the effective defensive strategy of the opposing team, lowering the possibilities of ways of getting the ball to them, or due to an ineffective capacity to create receiving opportunities in that game.

When analyzing the teams’ global rate of variability (Table 1), CH shows higher values than MC both for the passing (0.256 vs. −0.112) and the receiving (0.407 vs. 0.293) actions. It is important to note that the MC’s negative rate of total entropy depicts the team’s tendency to follow more stable passing patterns (out-entropy) between players than receiving (in-entropy) ones.

Regarding both teams’ index of capacity of passing (0.067 vs. 0.029) and receiving (0.107 vs. 0.077), the differences become clearer. CH showed a greater passing and receiving variability index. This shows that the team was more unpredictable in their actions, and thus more difficult to defend by the opponent, possibly creating better conditions to control the game and creating chances of winning it.

## 4. Conclusions

To summarize, using a mixed Bayesian and non-linear approach to network analysis, the proposed mathematical models allow us to better display the rate of variability within the passing sequences in the football game, increasing the tools available for match performance analysis. Higher rates of variability are associated to more unpredictability in the patterns, posing greater challenges to the opposing team. Additionally, the advantage of an index that allow one to analyze the performance on a finite scale provides the analysts with a clearer understanding of the team or player’s level of variability. Further studies should focus on applying these metrics to compare, for example, the effect of different tactical formations on the rate and index of variability of each player or the whole team. Additionally, comparing how these models behave against other metrics may be worthwhile, as the more tangible values given may be of greater value to the community. Finally, we hope to extend the uPATO platform to include these new mathematical models, making them available to the community and spreading their use and interpretation.

## Figures and Tables

**Table 1:** Team metrics of rate and index of variability.

	Chelsea	Manchester City
$C_{N}^{out}$	0.256	−0.112
$C_{N}^{in}$	0.407	0.293
$IndC_{N}^{out}$	0.067	0.029
$IndC_{N}^{in}$	0.107	0.077

## Data Availability

The data presented in this study are available on request from the corresponding author.

## Appendix A

**Table A1:** Individual passing and receiving capacity of variability.

	$R_{i}^{out}\left(n_{i}\right)$		$R_{i}^{in}\left(n_{i}\right)$	
Player	Chelsea	Manchester City	Chelsea	Manchester City
1	0.241	0.230	0.143	0.089
2	0.226	0.224	0.351	0.314
3	0.232	0.220	0.436	0.569
4	0.226	0.230	0.181	0.409
5	0.233	0.231	0.303	0.400
6	0.253	0.228	0.246	0.547
7	0.228	0.233	0.340	0.149
8	0.239	0.254	0.310	0.319
9	0.248	0.250	0.157	0.140
10	0.242	0.250	0.241	0.170
11	0.250	0.243	0.355	0.273
12	0.224	0.250	0.202	0.132
13	0.258	0.260	0.079	0.054
14	0.247	0.248	0.140	0.118

**Table A2:** Individual passing and receiving index of variability.

	$IndR_{i}^{out}\left(n_{i}\right)$		$IndR_{i}^{in}\left(n_{i}\right)$	
Player	Chelsea	Manchester City	Chelsea	Manchester City
1	0.063	0.061	0.038	0.023
2	0.059	0.059	0.092	0.082
3	0.061	0.058	0.115	0.149
4	0.059	0.061	0.048	0.108
5	0.061	0.061	0.080	0.105
6	0.066	0.060	0.065	0.144
7	0.060	0.061	0.089	0.039
8	0.063	0.067	0.081	0.084
9	0.065	0.066	0.041	0.037
10	0.064	0.066	0.063	0.045
11	0.066	0.064	0.093	0.072
12	0.059	0.066	0.053	0.035
13	0.068	0.068	0.021	0.014
14	0.065	0.065	0.037	0.031
